# Uncoupled Auxiliary S2-Alar-Iliac Screws in Long Lumbopelvic Constructs: Exploratory Finite Element Analysis and Preliminary Clinical Experience

**DOI:** 10.3390/jcm15197447

**Published:** 2026-09-24

**Authors:** Celal Ozbek Cakir, Murat Baloglu, Ulkun Unlu Unsal, Hakan Millet, Erdal Yayla, Eser Pala, Ismail Bozkurt

**Affiliations:** 1 Department of Neurosurgery, Afyon Park Hospital, Afyon 03100, Turkey; celal_ozbek@yahoo.com; 2 Department of Neurosurgery, Eskisehir City Hospital, Eskisehir 26080, Turkey; mbalogluog@gmail.com (M.B.); drhakanmillet@hotmail.com (H.M.); drerdalyayla08@gmail.com (E.Y.); 3 Department of Neurosurgery, Manisa City Hospital, Manisa 45040, Turkey; ulkununlu@hotmail.com; 4 MarlaDT Engineering, Adalet Mahallesi, Folkart Towers A Kule No:47/B, Bayrakli, Izmir 35530, Turkey; pala.eser@gmail.com; 5 Department of Neurosurgery, Faculty of Medicine, Yuksek Ihtisas University, Ankara 06530, Turkey; 6 Department of Neurosurgery, Medical Park Ankara Hospital, Ankara 06680, Turkey

**Keywords:** sacropelvic fixation, S2-alar-iliac (S2AI) screw, sacroiliac joint biomechanics, finite element analysis, adult spinal deformity

## Abstract

**Background and Objectives:** In this study, uncoupled auxiliary trans-sacroiliac (UATS) screws were evaluated as a mechanically independent adjunct to conventional S2-alar-iliac (S2AI) fixation in long lumbopelvic constructs. We assessed their exploratory biomechanical behavior and preliminary clinical feasibility. **Methods:** Conventional bilateral S2AI fixation and the same construct supplemented with bilateral UATS screws were compared in an exploratory finite element analysis under flexion, extension, lateral bending, and axial rotation. A retrospective clinical series of 26 patients undergoing UATS-supported lumbopelvic reconstruction was also evaluated. **Results:** Across the four loading modes, the UATS configuration showed lower global implant-system stress (81–92% reduction) and lower global sacral stress (43–67% reduction) within the computational model. The clinical cohort was revision dominant (25/26 patients). Mean SIJ-related pain VAS decreased from 8.0 ± 1.5 to 2.4 ± 1.0 and ODI from 68.1% ± 18.1% to 16.7% ± 8.6% at final follow-up (both *p* < 0.001). These clinical changes reflect the overall reconstructive procedure and cannot be attributed independently to UATS fixation. **Conclusions:** UATS placement was technically feasible in this small, revision-dominant cohort and altered stress distribution in an exploratory, unvalidated finite element model. The present study does not establish independent clinical efficacy or superiority over established pelvic fixation or SIJ fusion strategies.

## 1. Introduction

Long-segment thoracolumbar fusion to the pelvis is an established strategy for adult spinal deformity and complex lumbopelvic reconstruction. However, extending rigid spinal constructs to the pelvis substantially alters the biomechanics of the sacroiliac joint (SIJ) and may be associated with postoperative SIJ-related pain, implant-related complications, and mechanical failure at the lumbopelvic junction [1,2,3,4,5]. Conventional iliac and S2-alar-iliac (S2AI) screws provide robust distal fixation but expose the sacropelvic region to substantial mechanical demands.

Several strategies have been proposed to address the mechanical and clinical consequences of pelvic fixation, including additional pelvic fixation points, multirod constructs, and concurrent SIJ fixation or fusion [6,7,8,9]. One study demonstrated that concurrent SIJ fusion alongside S2AI fixation reduced postoperative SIJ pain [10]. Additional pelvic fixation may increase construct rigidity and improve resistance to selected modes of mechanical failure, although implant density and load distribution are altered by these strategies.

An alternative method involves providing supplemental trans-sacroiliac fixation without incorporating the additional screw into the longitudinal rod construct. In this configuration, referred to as an uncoupled auxiliary trans-sacroiliac (UATS) screw, the auxiliary implant traverses the sacrum and SIJ into the ilium but remains mechanically independent of the primary longitudinal fixation system. This configuration differs fundamentally from conventional S2AI fixation, dual iliac screw constructs, and multirod pelvic fixation, in which the pelvic implants are mechanically coupled to the longitudinal construct. It also differs from formal SIJ fusion because no intentional arthrodesis or bone-grafting procedure is performed across the SIJ. The theoretical rationale is that an independently positioned trans-SIJ implant may modify relative sacrum–ilium motion and local stress distribution while avoiding the intentional elimination of SIJ motion inherent to arthrodesis.

To our knowledge, the biomechanical behavior and early clinical experience of an uncoupled trans-SIJ screw used as an adjunct to S2AI fixation without intentional SIJ arthrodesis have not been systematically evaluated. We therefore performed a comparative finite element analysis (FEA) of conventional bilateral S2AI fixation with and without bilateral UATS screws and complemented the biomechanical analysis with a preliminary clinical series of patients undergoing complex lumbopelvic reconstruction. We hypothesized that the addition of UATS screws would alter stress distribution within the lumbopelvic construct and provide additional trans-SIJ stabilization without requiring intentional SIJ fusion.

## 2. Materials and Methods

### 2.1. Study Design and Patient Population

This study consisted of two complementary components: (1) a comparative FEA evaluating the biomechanical effect of adding UATS screws to conventional bilateral S2AI fixation and (2) a retrospective observational clinical series of patients undergoing long-segment lumbopelvic reconstruction incorporating UATS screws. We screened thirty-three consecutive patients who underwent UATS-supported lumbopelvic reconstruction at a single institution by a three-surgeon team. In total, 3 patients were excluded because the postoperative follow-up period was shorter than 14 months and 4 because the complete postoperative CT imaging required for radiographic assessment was unavailable, leaving 26 patients in the final cohort. The operative treatment period extended from January 2022 to January 2025. Clinical follow-up data available for the retrospective assessment extended through April 2026. Final follow-up was defined as each patient’s latest available clinical and radiographic assessment after satisfying the minimum 14-month follow-up requirement, rather than a predefined postoperative time point. Ultimately, 25 of the 26 patients underwent revision reconstruction. All patients had preoperative SIJ-related pain; however, SIJ pain was not itself the primary indication for the overall reconstructive surgery. UATS screws were used as an adjunct to conventional S2AI fixation in complex long lumbopelvic reconstructions and were not intended to achieve SIJ arthrodesis. Because no contemporaneous control group without UATS fixation was available, the clinical component was descriptive and was not designed to determine an independent causal effect of UATS screws. UATS fixation was not applied routinely to all revision cases. It was selected for patients with injection-confirmed preoperative SIJ-related pain and at least one additional mechanical-risk feature, including revision surgery, pseudarthrosis, implant failure, osteotomy requirement, a long construct, or poor bone quality; final use was determined by the surgeon’s intraoperative assessment. The period from January 2022 to January 2025 represents the period of clinical treatment and surgery rather than prospective enrollment into a research protocol. The clinical component of the present study was conducted as a retrospective review of previously treated patients.

### 2.2. Clinical and Radiological Evaluation

Preoperative SIJ-related pain was assessed using pain localization, physical examinations, and fluoroscopically guided SIJ injections. The Fortin finger finding was used to localize pain to the posterior superior iliac spine region and was not counted among the requirement for at least three positive SIJ provocation maneuvers. The provocation battery comprised the FABER, Gaenslen, thigh-thrust, pelvic compression, pelvic distraction, and sacral-thrust tests, with at least three positive maneuvers required. All patients additionally underwent fluoroscopically guided intra-articular SIJ injection 2–4 weeks before surgery. After intra-articular placement was confirmed with a small amount of contrast, 1 mL of 0.25% bupivacaine plus 1 mL of triamcinolone acetonide 40 mg/mL (total volume, 2 mL) was administered. A positive diagnostic response was defined as at least a 50% reduction in visual analogue scale (VAS) pain during standard SIJ provocation maneuvers 30 min after injection, thereby assessing the early local anesthetic response separately from the delayed corticosteroid effect. All 26 patients fulfilled these preoperative diagnostic criteria. Clinical outcomes included lower-back pain and SIJ-related pain, measured using a VAS, together with disability, assessed using the Oswestry Disability Index (ODI). Because the study was retrospective, the complete provocation test battery was not systematically repeated at every postoperative visit; therefore, absence of a documented positive postoperative finding was not interpreted as a systematically confirmed negative examination. Patient satisfaction at final follow-up was abstracted retrospectively from the clinical records using the documented categories “satisfied” and “extremely satisfied”; no prospectively administered validated satisfaction instrument was used.

Radiographic evaluation included standing spinal radiographs and computed tomography (CT). All 26 patients underwent an early postoperative CT before discharge for assessment of implant position and a 12-month CT for radiographic follow-up. CT examinations were independently reviewed by two spine surgeons who were blinded to the clinical outcomes. Lumbosacral fusion was assessed primarily at L5–S1. Solid fusion was defined as continuous trabecular bridging through or around the interbody space and/or continuous posterolateral bridging bone without a persistent radiolucent line. Peri-implant radiolucency was defined as a radiolucent zone at least 1 mm wide at the bone–screw interface, visible on at least two consecutive CT slices and confirmed in two orthogonal planes. Imaging was also reviewed for screw position, migration, pullout, breakage, cortical breach, rod failure, and postoperative SIJ bridging or ankylosis. Spinopelvic parameters, including lumbar lordosis (LL), pelvic incidence–lumbar lordosis mismatch (PI–LL), and sagittal vertical axis (SVA), were assessed preoperatively and at final follow-up.

### 2.3. Surgical Technique

Patients were positioned prone, and the posterior elements of the lumbosacral spine were exposed according to the requirements of the reconstruction. Following identification of the S1 and S2 dorsal foramina, the primary S2AI screw was placed using a modified entry point approximately 1–2 mm inferior and 1–2 mm lateral to the S1 dorsal foramen (upper screw entry point in Figure 1a). Under fluoroscopic guidance, the screw trajectory was directed 30–40° laterally and 20–30° caudally, with the caudal angulation adjusted according to the individual sacral slope. A standard 8 mm × 80 mm S2AI screw was then inserted (Aero Medical Thoracolumbar Stabilization System, Ankara, Türkiye). 

The UATS screw entry point was located approximately 1 mm superior and 1 mm lateral to the S2 dorsal foramen (lower screw entry point in Figure 1a). Its trajectory followed a lateral angulation similar to that of the primary S2AI screw but with slightly less caudal angulation to avoid the sciatic notch (Figure 1b,c). Under fluoroscopic guidance using teardrop views (Figure 1d), an 8 mm × 80 mm screw was advanced across the SIJ into the ilium, with attention paid to maintaining an adequate osseous corridor and avoiding cortical breach. The inter-screw distance (approximately 5 mm) was defined as the minimum edge-to-edge distance between the outer surfaces of the primary S2AI and UATS screws at the point where the two trajectories came closest within the sacroiliac osseous corridor; it was not a center-to-center measurement. This intervening osseous bridge was preserved to reduce trajectory overlap. A representative postoperative three-dimensional CT reconstruction demonstrating the relative trajectories of the primary S2AI and UATS screws across the sacroiliac region is shown in Figure 1e.

The UATS screw was inserted using the manufacturer-approved tulip-less configuration and seated using the dedicated driver. The screw was intentionally left mechanically uncoupled from the longitudinal rod and multirod construct. Thus, unlike the primary S2AI screw, the UATS screw had no direct connection to the longitudinal fixation system. No bone graft or other procedure intended to achieve SIJ arthrodesis was performed as part of the UATS screw placement. No intraoperative iliac fracture related to placement of the adjacent pelvic screws was observed in this cohort.

### 2.4. Statistical Analysis

Continuous variables were summarized using the mean ± standard deviation (SD) when approximately normally distributed and median with interquartile range (IQR) when distributions were non-normal. Categorical variables were summarized as frequencies and percentages. For longitudinal clinical and radiographic outcomes, patient-level paired data were used for preoperative-to-final-follow-up comparisons. The distribution of within-patient differences was assessed using the Shapiro–Wilk test. Paired-samples *t* tests were used when paired differences were approximately normally distributed, whereas Wilcoxon signed-rank tests were used for non-normally distributed paired differences. For normally distributed paired outcomes, mean changes and corresponding 95% confidence intervals (CIs) were calculated. For non-normally distributed outcomes, medians and IQRs were emphasized. Intermediate postoperative time points were summarized descriptively; inferential testing was restricted to prespecified paired comparisons between preoperative and final follow-up values. All statistical tests were two-sided, and a *p* value < 0.05 was considered statistically significant. Because the clinical component was an uncontrolled observational series, statistical comparisons represent within-cohort changes following the overall lumbopelvic reconstruction and should not be interpreted as estimates of an independent treatment effect attributable to UATS fixation. No adjustment for multiple comparisons was performed; therefore, secondary outcome analyses were considered exploratory. Minimum clinically important difference (MCID) responder analyses were not performed because outcome-specific MCID thresholds had not been prespecified in the study protocol. Statistical analyses were performed using IBM SPSS Statistics, version 29 (IBM Corp., Armonk, NY, USA).

### 2.5. Finite Element Model Construction and Material Properties

The computational analysis used a pre-existing three-dimensional CAD model of the lower lumbar spine and pelvis obtained from GrabCAD (Human Lumbar Spine 2; https://grabcad.com/library/human-lumbar-spine-2, (accessed on 1 February 2026)). Because a ready CAD model was used, no CT/DICOM segmentation was performed. The analyzed geometry comprised L4, L5, the sacrum, and the pelvis, and it therefore represented the distal lumbopelvic segment rather than the full length of the clinical constructs. Surface reconstruction/repair, geometry cleanup, and implant placement and positioning were performed in ANSYS SpaceClaim 2022 R2 (Version 22.2). Material assignment, meshing, contact definition, boundary-condition definition, solution, and post-processing were performed in ANSYS Mechanical/Workbench 2022 R2 (Version 22.2). Posterior instrumentation extended from L4 to the pelvis, and no spinal implants other than the modeled screws and rods were included.

The final finite element model comprised 1,369,797 nodes and 2,966,965 elements. Second-order 10-node tetrahedral SOLID187 elements were used for the modeled bone, cartilage, and implant geometries, with a final element size of approximately 1–2 mm. Bone, cartilage, and metallic implant components were modeled as homogeneous, isotropic, linear-elastic materials. This constitutive simplification was applied identically to both fixation configurations and was intended for comparative rather than patient-specific physiological prediction.

All interacting interfaces in the final analyzed model were idealized as bonded, including the bone–screw, screw–rod, and SIJ articular interfaces. Thus, separation and sliding at these interfaces were neglected. Sacroiliac ligament structures were not explicitly represented in the model. These assumptions were applied identically to both fixation configurations.

Material properties were selected from previously published finite element models of the pelvis and SIJ [11,12,13,14]. Cortical bone was assigned a Young’s modulus of 12,000 MPa and Poisson’s ratio of 0.30; cancellous bone, 100 MPa and 0.20; SIJ articular cartilage, 100 MPa and 0.30; pubic symphysis, 5 MPa and 0.45; vertebral endplate, 1000 MPa and 0.40; and titanium-alloy fixation components, 110,000 MPa and 0.30. The same material definitions were used in both fixation configurations (Table 1).

Bone, cartilage, and metallic implant components were modeled as homogeneous, isotropic, linear-elastic materials. Material properties were applied identically in both fixation configurations. Titanium-alloy properties correspond to Ti-6Al-4V-type orthopedic implant material. Values were selected from previously published finite element models of the pelvis and sacroiliac region.

#### 2.5.1. Boundary Conditions, Loading, and Fixation Configurations

The model was evaluated under flexion, extension, lateral bending, and axial rotation using identical boundary and loading conditions for both fixation configurations. For each loading mode, a 400 N follower load and a 7.5 Nm directional moment were applied through a reference point located at the superior endplate of L4, the uppermost modeled vertebra. The reference point was coupled to the superior endplate using rigid body/kinematic coupling (RBE2), distributing the applied load across the endplate surface rather than concentrating it at a single node (Figure 2).

**Figure 2:**
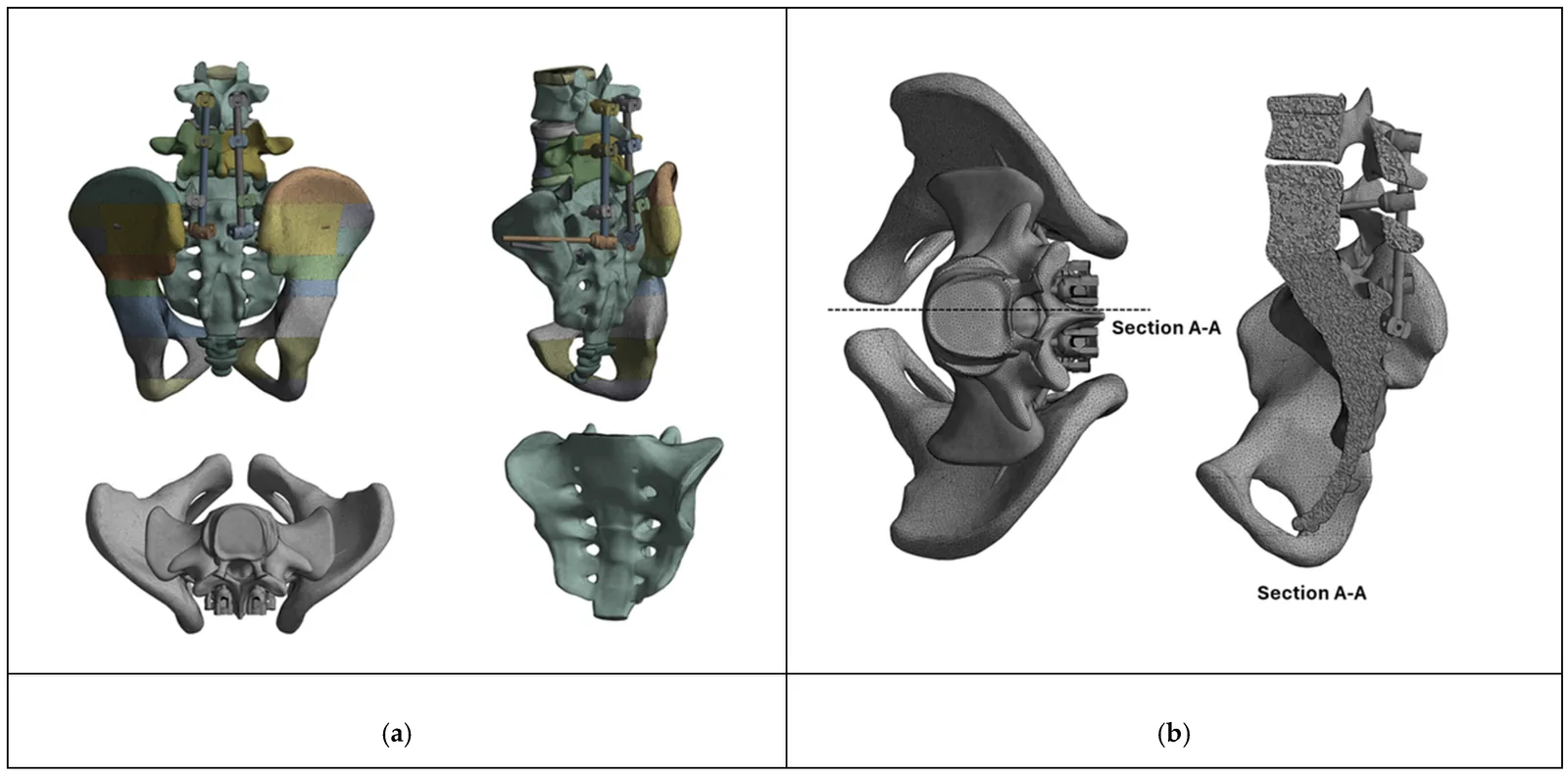

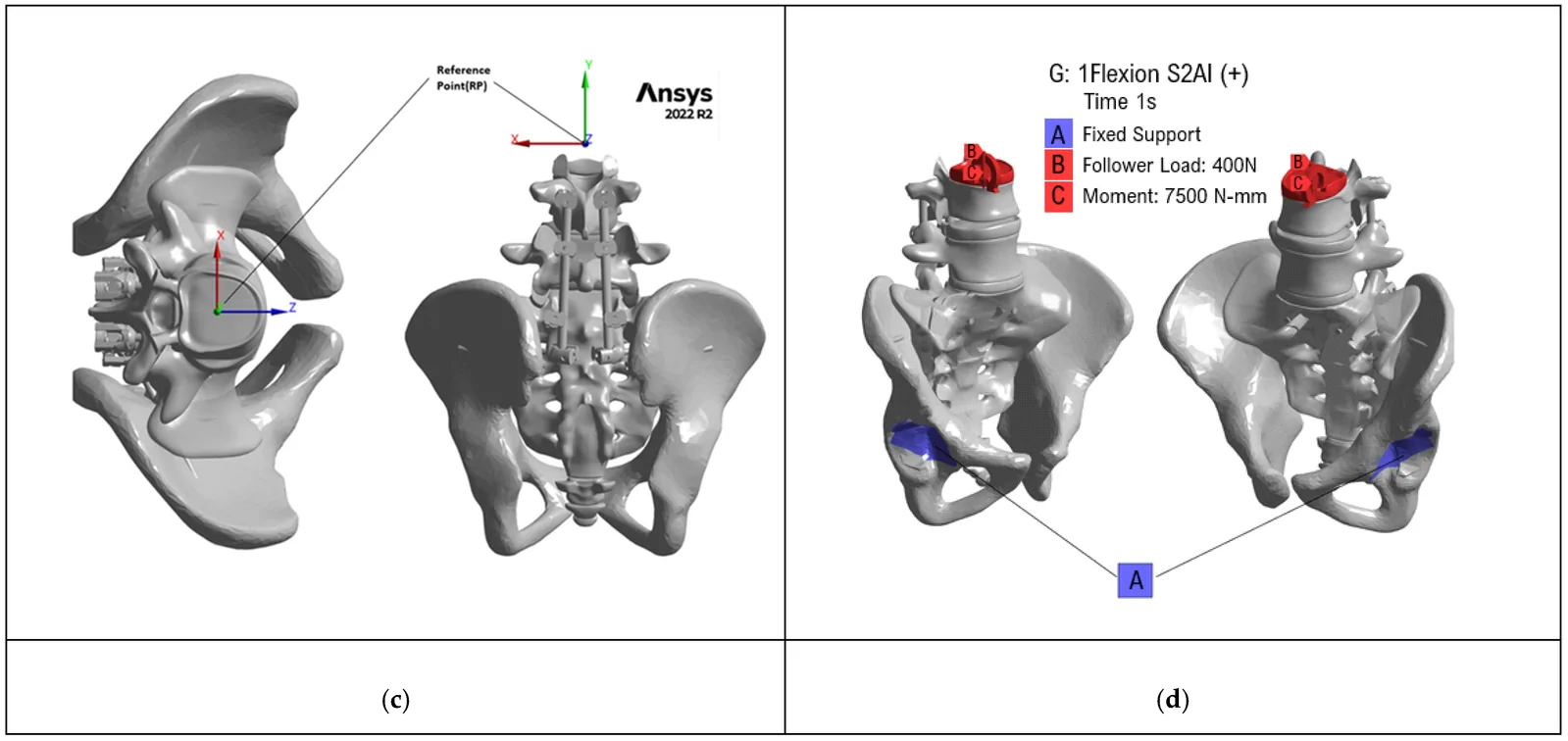
Direct exports from the actual ANSYS Workbench 2022 R2 finite element model. (**a**) Modeled lumbopelvic geometry and instrumentation; (**b**) finite element mesh/cross-sectional view; (**c**) reference point (RP) used for load application at the superior endplate of L4; and (**d**) loading and boundary-condition view showing the 400 N follower load, 7.5 Nm moment, and bilateral acetabular fixed supports. Figure 2 documents the computational setup, whereas Figure 3 presents a representative direct ANSYS result output.

**Figure 3:**
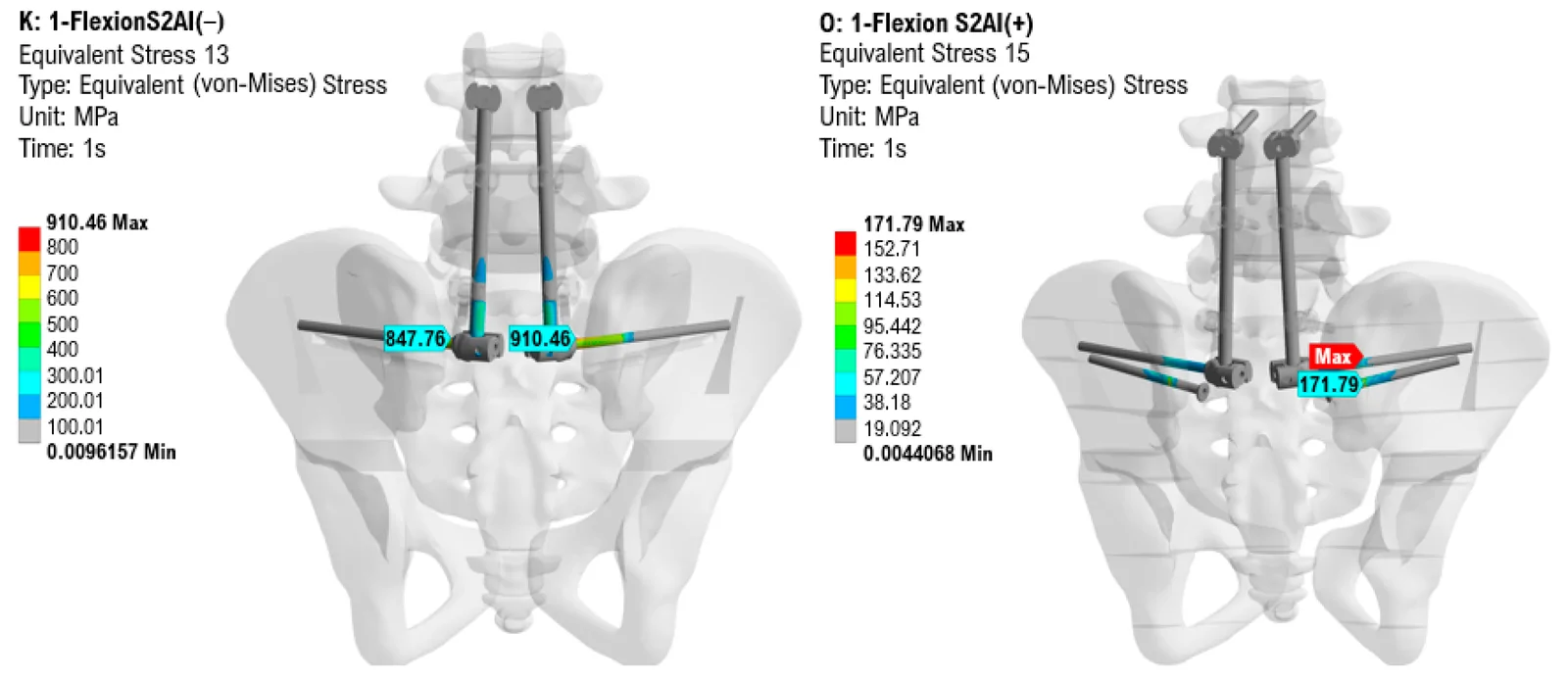
Representative direct ANSYS output under flexion showing global von Mises stress in the combined screw–rod implant system for the S2AI(−) and S2AI(+) configurations. The displayed maxima are approximately 910.46 MPa and 171.79 MPa, respectively. Complete mode-specific numerical outputs are provided in Table 2.

The bilateral acetabular regions were constrained in all six degrees of freedom (Ux = Uy = Uz = 0; Rx = Ry = Rz = 0). The sacrum and SIJ were not directly constrained, allowing for the transfer of the applied loads through the lumbopelvic construct to the sacrum and pelvis under the specified boundary conditions (Figure 2d).

Two fixation configurations were compared. In the comparator configuration [S2AI(−)], conventional bilateral S2AI screws were connected to the longitudinal rod system. In the augmented configuration [S2AI(+)], the same bilateral S2AI construct was supplemented with two additional trans-SIJ UATS screws. The UATS screws were not connected to the longitudinal rods or connectors and therefore had no direct mechanical coupling to the longitudinal rod construct. Within the finite element idealization, the auxiliary screws acted as trans-SIJ fixation elements through the bonded bone–screw interfaces.

#### 2.5.2. Model Validation and Numerical Interpretation

The finite element framework was interpreted as an exploratory comparative computational model rather than as an independently validated predictor of absolute in vivo SIJ stresses or kinematics. Previous SIJ finite element studies have shown that estimated motion and local stress are sensitive to boundary conditions, material definitions, mesh characteristics, and contact formulation [11,12,13,14].

A mesh-refinement assessment was performed during model development by progressively reducing the characteristic element size from approximately 4 mm to 3 mm and then to 2 mm, with refinement stopped when total deformation was observed to remain stable. However, mesh-specific node/element counts and percentage changes in the principal outputs were not retained; therefore, this assessment was not treated as a formal quantitative mesh convergence study. A contact sensitivity analysis using alternative contact formulations was not performed, and the model was not quantitatively validated against an experimental, cadaveric, in vivo, or published physiological benchmark. These limitations were considered when interpreting the results.

Matched numerical outputs were available for flexion, extension, lateral bending, and axial rotation. Principal comparisons were based on global component maxima for total deformation and von Mises stress in the sacrum, SIJ cartilage, ilium, and combined screw–rod implant system. Local probe values shown on selected engineering contour maps were not used interchangeably with the global component maxima. Because the archived implant output treated screws and rods as a single titanium implant group, the exact implant subcomponent at which the global maximum occurred could not be assigned reliably.

## 3. Results

### 3.1. Finite Element Analysis

Matched analyses were available for all four loading modes. Relative to the conventional S2AI configuration [S2AI(−)], the UATS-augmented configuration [S2AI(+)] showed lower total deformation (94–95% reduction), lower global sacral von Mises stress (43–67% reduction), lower SIJ cartilage stress (72–90% reduction), lower combined screw–rod implant-system stress (81–92% reduction), and lower iliac stress (49–83% reduction) across the tested loading modes (Table 2). The implant-system output represents the combined screws and rods rather than a rod-only result, and the exact implant subcomponent containing the global maximum was not separately resolved. Because no formal quantitative mesh convergence table, contact sensitivity analysis, or independent validation benchmark was available, these values were interpreted as exploratory comparative outputs rather than direct physiological measurements.

Values are global component outputs from the final ANSYS (Version 22.2) analysis. The implant-system output comprises screws and rods analyzed as one titanium implant group. Local probe values shown in selected contour plots are not substituted for the global component maxima.

### 3.2. Patient Demographics and Surgical Characteristics

The final clinical cohort comprised 26 patients, including 21 women (80.8%) and 5 men (19.2%), with a mean age of 69.8 ± 8.1 years. Twenty-five patients (96.2%) underwent revision reconstruction, whereas one patient (3.8%) underwent index surgery. Mean clinical follow-up was 14.7 months (range, 14–18 months). Bone mineral density T-scores ranged from 0 to −3.0 (Table 3). Proximal instrumented levels ranged from T2 to L2, with all constructs extending distally to the pelvis. All patients underwent posterior column osteotomy (PCO), and 10 of 26 patients (38.5%) additionally underwent pedicle subtraction osteotomy (PSO). Mean operative duration was 4.9 h, and mean estimated blood loss was 1461.5 mL (Table 4). Revision indications included pseudarthrosis, proximal junctional failure, adjacent segment disease, and implant failure. Three patients (#9, #18, and #23) had previously undergone iliac fixation; the prior iliac implants were removed during revision reconstruction and replaced with the pelvic fixation configuration used in the present study.

### 3.3. Clinical Outcomes

Pain and disability measures improved between the preoperative and final follow-up assessments (Table 5). Mean lower-back VAS decreased from 7.2 ± 1.4 preoperatively to 1.8 ± 0.8 at final follow-up (Wilcoxon signed-rank test, *p* < 0.001). Mean SIJ-related pain VAS decreased from 8.0 ± 1.5 to 2.4 ± 1.0 (Wilcoxon signed-rank test, *p* < 0.001). Mean ODI decreased from 68.1% ± 18.1% preoperatively to 16.7% ± 8.6% at final follow-up, corresponding to a mean paired reduction of 51.3 percentage points (95% CI, 44.7–57.9; paired *t* test, *p* < 0.001). Postoperative SIJ provocation testing was not standardized at every visit; therefore, absence of a recorded positive provocation finding was not interpreted as a systematically confirmed negative examination. Ultimately, 11 of 26 patients (42.3%) were recorded as extremely satisfied and 15 of 26 (57.7%) as satisfied at final follow-up. These findings represent within-cohort changes after the complete reconstructive procedure and cannot be attributed independently to UATS fixation.

### 3.4. Radiographic Outcomes and Spinopelvic Alignment

All 26 patients had both early postoperative CT and 12-month CT imaging. At 12 months, 23 of 26 patients met the predefined CT criteria for solid L5–S1 fusion. Patients #1, #2, and #12 did not meet the criteria for solid fusion and were classified as indeterminate rather than as definite pseudarthrosis. Radiolucent halos were identified around a primary S2AI screw in four patients (#1, #12, #13, and #21; 15.4% on a patient-level basis) and around a UATS screw in one patient (#2; 3.8%). The corresponding T-scores for halo patients #1, #2, #12, #13, and #21 were −1.1, −2.5, −2.0, −1.5, and −1.0, respectively. Patients #1, #2, and #12 had indeterminate fusion assessments; Patients #13 and #21 met the fusion criteria, and pelvic screw-region pain was documented in Patients #13 and #21. No screw migration, pullout, breakage, or cortical breach was identified. Although SIJ arthrodesis was not intended, focal or partial SIJ bridging was observed in two patients and complete continuous bilateral SIJ ankylosis in one patient; degenerative SIJ changes such as sclerosis, osteophytes, or joint-space narrowing were observed in approximately 60% of the cohort. These findings are reported descriptively without formal between-implant comparison.

Spinopelvic alignment improved between the preoperative and final follow-up assessments. Mean lumbar lordosis increased from 40.7° to 49.0° (Wilcoxon signed-rank test, *p* < 0.001), and mean PI–LL mismatch decreased from 17.6° to 9.3° (Wilcoxon signed-rank test, *p* < 0.001). After correction of the Patient #26 SVA entries to match the original Figure 4 annotations (82.3 and 80.8 mm), mean SVA decreased from 5.80 ± 1.26 cm preoperatively to 5.10 ± 1.12 cm at final follow-up, corresponding to a mean paired reduction of 0.70 cm (95% CI, 0.31–1.09; paired *t* test, *p* < 0.001) (Figure 4 and Table 6). These alignment changes reflect the overall reconstructive procedure and were not interpreted as an effect attributable specifically to UATS fixation.

### 3.5. Complications

Two patients (#9 and #26) developed surgical-site infections; both were successfully treated with intravenous antibiotics without surgical debridement or implant removal. No wound dehiscence, postoperative hematoma requiring intervention, new neurological deficit, vascular injury, iliac fracture, screw malposition or cortical breach, screw migration, rod fracture, proximal junctional failure, major medical complication, symptomatic pseudarthrosis, or reoperation for any cause was documented during follow-up. The clinical and radiographic outcomes recorded in the last follow-up are summarized in Table 7.

## 4. Discussion

This study combines an exploratory comparative FEA with a preliminary clinical series of UATS-supported lumbopelvic reconstruction. In the computational model, adding UATS screws was associated with lower total deformation and lower global sacral, SIJ cartilage, iliac, and combined screw–rod implant-system stresses across flexion, extension, lateral bending, and axial rotation. These findings indicate altered modeled load transfer under the specified assumptions, but the model was not validated against experimental, cadaveric, in vivo, or published physiological benchmark data. Contact sensitivity analysis was not performed, and the available mesh refinement assessment did not provide a retained quantitative convergence dataset. The FEA findings should therefore be interpreted as exploratory comparative observations rather than evidence of an in vivo mechanical advantage [11,12,13,14].

The clinical component was small, uncontrolled, and strongly revision dominant: 25 of 26 patients underwent revision reconstruction, frequently together with osteotomy, interbody reconstruction, decompression, deformity correction, and extension of prior instrumentation. UATS placement was technically feasible within these complex procedures, but the observed improvements in VAS, ODI, and alignment reflect the overall reconstructive operation and cannot be attributed independently to UATS fixation. The present clinical data therefore do not establish efficacy, prevention of SIJ pain, or superiority over conventional S2AI fixation, multirod constructs, or SIJ fusion.

All patients underwent early postoperative and 12-month CT, with the 12-month studies independently assessed by two spine surgeons blinded to clinical outcomes. Twenty-three patients met the predefined CT criteria for solid L5–S1 fusion and three were classified as indeterminate rather than definite pseudarthrosis. Primary S2AI radiolucency was observed in four patients and UATS radiolucency in one patient. These findings are descriptive only because the screw types have different mechanical roles and the retrospective imaging review was not designed as a standardized screw-level comparison. Although SIJ arthrodesis was not intended, focal or partial bridging was observed in two patients and complete bilateral SIJ ankylosis in one, underscoring the distinction between surgical intent and postoperative joint status.

The concept of augmenting the pelvic foundation with additional fixation points has been examined using multiple pelvic screws, multirod constructs, and other augmented strategies [6,7,8,9]. Finite element work has shown that such configurations can alter lumbosacral instrumentation stresses [6]. UATS fixation differs mechanically because the auxiliary trans-SIJ screws remain uncoupled from the longitudinal rod system and are placed without an intended arthrodesis procedure. This distinction supports further comparative investigation but does not establish equivalence or superiority to established augmentation strategies. Prior clinical observations also suggest that implant behavior across the SIJ may influence postoperative symptoms, although the relationship between fixation characteristics and SIJ pain remains incompletely defined [15].

Placement of a second trans-SIJ screw adjacent to the primary S2AI trajectory requires adequate sacropelvic bone stock and careful trajectory control. In the present technique, the approximately 5 mm separation was defined as the minimum edge-to-edge distance between the outer screw surfaces at their closest point within the sacroiliac osseous corridor. No cortical breach, iliac fracture, or clinically apparent neurovascular injury related to UATS placement was documented during the available follow-up. Nevertheless, these observations from a small series should not be interpreted as establishing the safety profile of the technique; careful preoperative CT assessment and fluoroscopic or navigated trajectory control remain important, particularly in constrained osseous corridors [16].

Several limitations are central to interpretation. The clinical study was retrospective, uncontrolled, heterogeneous, and limited to short-term follow-up, with no contemporaneous non-UATS comparator. The radiographic assessment was retrospective, and halo findings were descriptive. The FEA idealized all interacting interfaces as bonded, did not explicitly model SIJ ligaments, did not incorporate patient-specific bone quality or biological remodeling, lacked formal contact-sensitivity testing, and was not independently validated against physiological or experimental benchmarks. Accordingly, the present findings are hypothesis-generating ones. Validated comparative biomechanical studies and prospective controlled clinical cohorts are required to determine whether UATS fixation provides any clinically meaningful advantage and to define its indications, long-term implant behavior, and relationship to SIJ-specific outcomes.

## 5. Conclusions

UATS placement was found to be technically feasible in this small, revision-dominant cohort, and the addition of UATS fixation altered stress distribution and deformation in an exploratory, unvalidated finite element model. The present study does not establish independent clinical efficacy, the preservation of physiological SIJ motion, the prevention of SIJ pain, reductions in implant failure, or superiority over standard S2AI fixation, multirod constructs, or SIJ fusion. Validated comparative biomechanical studies and controlled prospective clinical cohorts are required before such conclusions can be drawn.

## Figures and Tables

**Figure 1:**
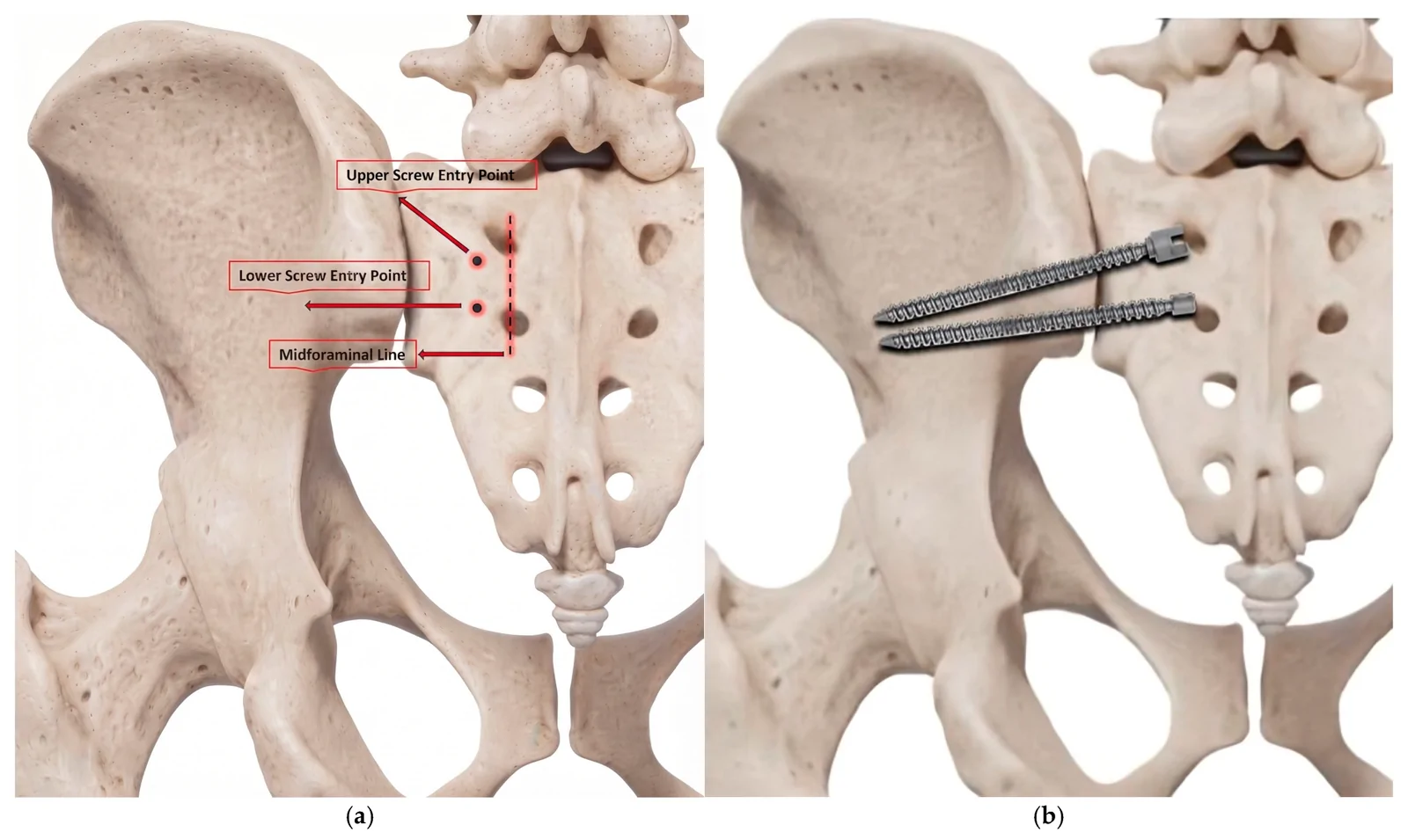

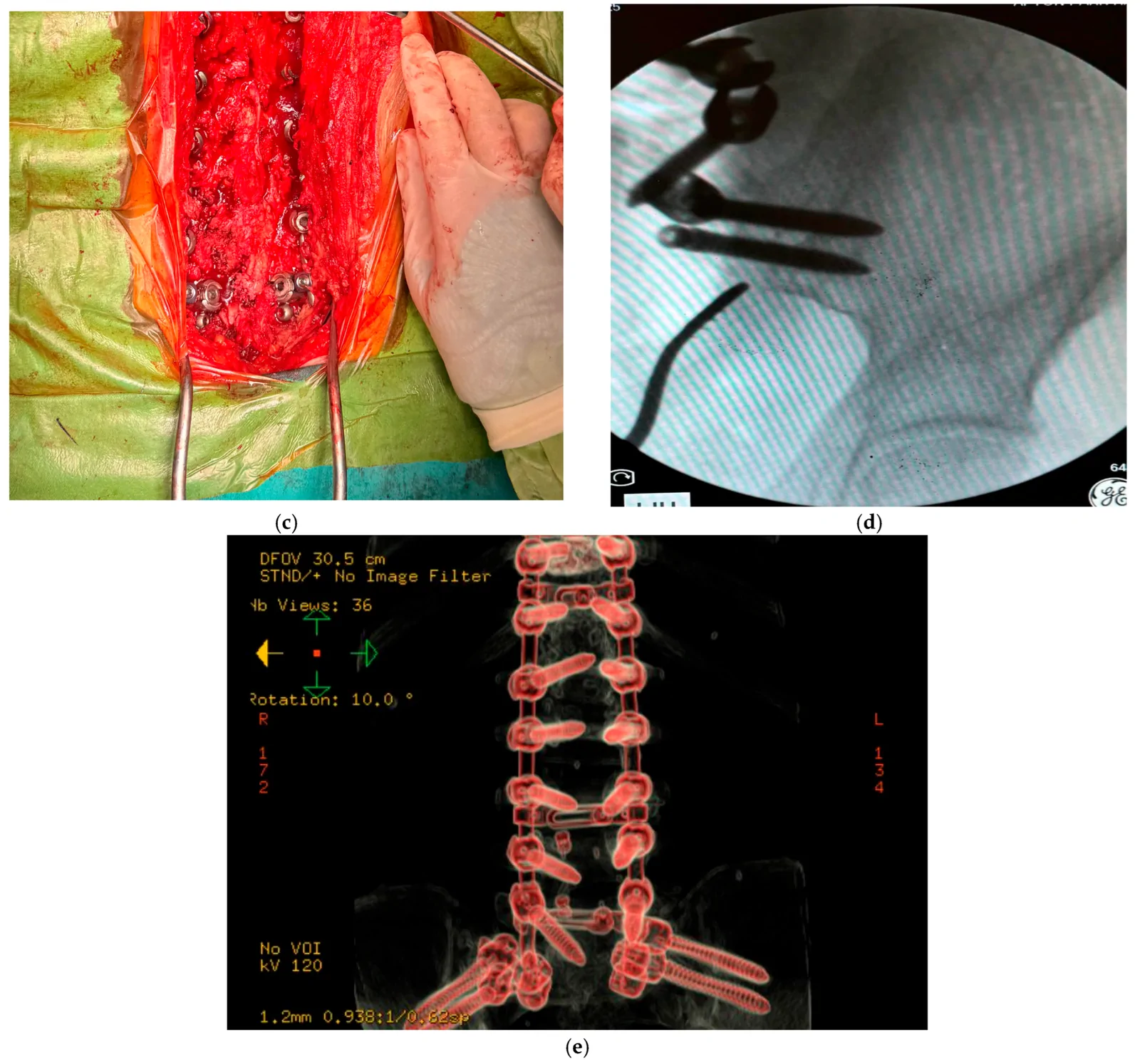
Surgical technique for UATS screw placement. (**a**) Anatomical landmarks demonstrating the entry points of the primary S2AI (upper screw entry point) and UATS (lower screw entry point) screws. (**b**) Final positioning of the primary S2AI and UATS screws across the sacroiliac joint. The approximately 5 mm separation refers to the minimum edge-to-edge distance between the outer screw surfaces at their closest point within the sacroiliac osseous corridor. (**c**) Intraoperative view of screw placement. (**d**) Intraoperative view illustrating that the UATS screw remains mechanically uncoupled from the longitudinal rod construct. (**e**) Representative anonymized postoperative three-dimensional CT reconstruction demonstrating the relative trajectories of the primary S2AI and UATS screws across the sacroiliac region.

**Figure 4:**
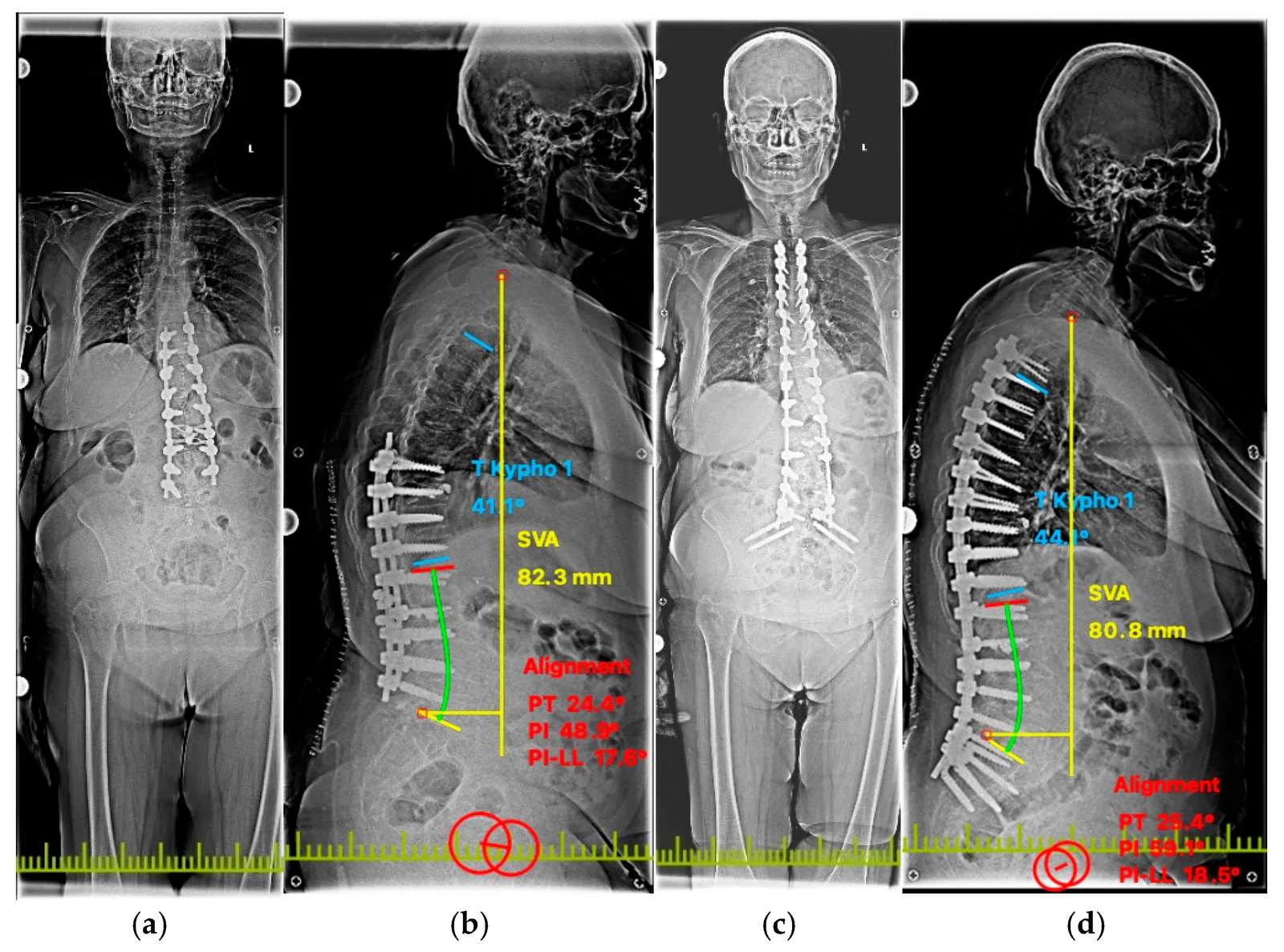
Representative preoperative and postoperative radiographs of Patient #26. (**a**) Preoperative anteroposterior standing radiograph demonstrating the underlying spinal deformity. (**b**) Preoperative lateral standing radiograph demonstrating sagittal malalignment; SVA is 82.3 mm. (**c**) Postoperative anteroposterior standing radiograph demonstrating the final instrumented construct. (**d**) Postoperative lateral standing radiograph following deformity correction and lumbopelvic reconstruction; SVA improved to 80.8 mm.

**Table 1:** Material properties used in the finite element model.

Component	Young’s Modulus/Stiffness	Poisson’s Ratio	Element/Material Representation
Sacral cortical bone	12,000 MPa	0.30	Homogeneous, isotropic, linear elastic
Sacral cancellous bone	100 MPa	0.20	Homogeneous, isotropic, linear elastic
Iliac cortical bone	12,000 MPa	0.30	Homogeneous, isotropic, linear elastic
Iliac cancellous bone	100 MPa	0.20	Homogeneous, isotropic, linear elastic
Vertebral cortical bone	12,000 MPa	0.30	Homogeneous, isotropic, linear elastic
Vertebral cancellous bone	100 MPa	0.20	Homogeneous, isotropic, linear elastic
SIJ articular cartilage	100 MPa	0.30	Homogeneous, isotropic, linear elastic
Pubic symphysis	5 MPa	0.45	Homogeneous, isotropic, linear elastic
Vertebral endplate	1000 MPa	0.40	Homogeneous, isotropic, linear elastic
Titanium-alloy screws/rods	110,000 MPa	0.30	Homogeneous, isotropic, linear elastic

**Table 2:** Mode-specific finite element outputs for the conventional S2AI and UATS-augmented configurations.

Load Case	Output	S2AI(−)	S2AI(+)	Change
Flexion	Total deformation (mm)	14.632	0.88189	−94%
	Sacrum stress (MPa)	136	77	−43%
	SIJ cartilage stress (MPa)	12.033	1.1569	−90%
	Implant-system stress (MPa)	910	171.79	−81%
	Ilium stress (MPa)	82	42	−49%
Extension	Total deformation (mm)	4.1542	0.20933	−95%
	Sacrum stress (MPa)	39	13	−67%
	SIJ cartilage stress (MPa)	3.4139	0.88722	−74%
	Implant-system stress (MPa)	431	34.1	−92%
	Ilium stress (MPa)	30	5	−83%
Lateral bending	Total deformation (mm)	9.4562	0.50754	−95%
	Sacrum stress (MPa)	88	41	−53%
	SIJ cartilage stress (MPa)	7.7809	2.2039	−72%
	Implant-system stress (MPa)	803	94.5	−88%
	Ilium stress (MPa)	43	22	−49%
Axial rotation	Total deformation (mm)	9.4772	0.52539	−94%
	Sacrum stress (MPa)	87	44	−49%
	SIJ cartilage stress (MPa)	8.4989	1.83	−78%
	Implant-system stress (MPa)	701	91.8	−87%
	Ilium stress (MPa)	49	23	−53%

**Table 3:** Patient demographics.

Patient No	Age	Sex	BMI	Diabetes	Tobacco	Surgical Status	BMD (T Score)	Previous Surgery	Follow-up (Months)
1	72	M	27	-	-	Revision	−1.1	1 time, L2-S1 fusion	14
2	71	F	29	-	-	Revision	−2.5	1 time, L1-S1 fusion	14
3	78	F	21	Type 2	-	Revision	−2.8	1 time, T11-L5 fusion	14
4	76	M	20	-	+	Revision	−1.5	1 time, T12-S1 fusion	14
5	82	F	25	Type 2	-	Revision	−3.0	2 times, T12-S1 fusion then second surgery removal of hardware	15
6	75	F	28	Type 2	-	Revision	−2.5	1 time, L1-S1 fusion	14
7	74	F	25	-	-	Revision	−2	1 time, L1-L5 fusion	14
8	66	F	19	-	-	Revision	−1	1 time, T12-S1 fusion	18
9	73	F	26	-	-	Revision	−2	2 times, T9-Iliac fusion	14
10	55	F	20	-	-	Revision	−1.5	1 time, T10-S1 fusion	14
11	77	M	21	-	-	Revision	−1.5	2 times, L5-S1 fusion	14
12	71	F	28	Type 2	-	Revision	−2	1 time, L2-L5 fusion	15
13	54	F	30	Type 2	-	Revision	−1.5	2 times, L2-S1 fusion	14
14	74	F	19	-	-	Revision	−2.5	3 times, T12-S1 fusion	14
15	75	F	28	Type 2	-	Revision	−2.5	1 time, L2-L5 fusion	16
16	65	F	26	-	-	Revision	−1.5	1 time, L3-S1 fusion	16
17	65	F	20	-	-	Revision	−1	1 time, L2-L5 fusion	15
18	76	M	22	-	+	Revision	−1.5	2 times, T12-Iliac fusion	14
19	77	M	19	-	+	Revision	−1	1 time, L1-S1 fusion	14
20	64	F	26	-	-	Revision	−1.5	1 time, L2-S1 fusion	14
21	56	F	29	Type 2	-	Index Surgery	−1	-	16
22	62	F	19	-	-	Revision	−1.5	2 times, L1-S1 fusion	14
23	69	F	28	-	-	Revision	−2.5	2 times, L2-Iliac fusion	14
24	79	F	19	-	-	Revision	−3	1 time, L2-S1 fusion	14
25	55	F	26	-	-	Revision	0	1 time, L2-L5 fusion	15
26	75	F	25	Type 2	-	Revision	−3	2 times, T9-L5 fusion	17

*BMI: Body Mass Index, BMD: Bone Mineral Density. Surgical Status indicates revision versus index surgery. Patients 9, 18, and 23 had prior iliac fixation.*

**Table 4:** Surgical details.

Patient No	Fusion Level	Interbody Fusion (PEEK Cage)	Osteotomy	Operation Duration (h)	EBL (cc)
1	L2-Pelvis	L3-L4 L4-L5 L5-S1	3 level PCO 1 level PSO	3	750
2	T9-Pelvis	L5-S1	4 level PCO	5	1500
3	T9-Pelvis	L4-L5 L5-S1	4 level PCO	5	1250
4	T9-Pelvis	L3-L4 L5-S1	3 level PCO 1 level PSO	6	2000
5	L1-Pelvis	L5-S1	2 level PCO 1 level PSO	3	1200
6	T9-Pelvis	L3-L4 L5-S1	3 level PCO 1 level PSO	5	2000
7	T4-Pelvis	L5-S1	5 level PCO	6	2200
8	T10-Pelvis	L5-S1	3 level PCO	4	1500
9	T8-Pelvis	L2-L3 L3-L4 L4-L5 L5-S1	3 level PCO 1 level PSO	6	1500
10	T10-Pelvis	L3-L4 L4-L5 L5-S1	3 level PCO	5	1250
11	T12-Pelvis	L3-L4 L4-L5 L5-S1	3 level PCO	5	1500
12	T12-Pelvis	L3-L4 L4-L5 L5-S1	3 level PCO 1 level PSO	6	1200
13	L1-Pelvis	L4-L5 L5-S1	3 level PCO	4	750
14	T10-Pelvis	L3-L4 L4-L5 L5-S1	3 level PCO 1 level PSO	6	1600
15	T12-Pelvis	L4-L5 L5-S1	3 level PCO	4	1250
16	L1-Pelvis	L3-L4 L4-L5 L5-S1	3 level PCO	5	1500
17	T12-Pelvis	L4-L5 L5-S1	4 level PCO	5	1250
18	T10-Pelvis	L3-L4 L4-L5 L5-S1	3 level PCO 1 level PSO	6	2000
19	T10-Pelvis	L4-L5 L5-S1	3 level PCO	5	800
20	T12-Pelvis	L5-S1	3 level PCO	4	750
21	L1-Pelvis	L3-L4 L4-L5 L5-S1	3 level PCO 1 level PSO	5	2000
22	T10-Pelvis	L4-L5 L5-S1	4 level PCO	5	1750
23	T12-Pelvis	L3-L4 L4-L5 L5-S1	4 level PCO	4	1250
24	T10-Pelvis	L4-L5 L5-S1	4 level PCO	5	1750
25	T12-Pelvis	L4-L5 L5-S1	3 level PCO	4	1000
26	T2-Pelvis	None	5 level PCO 1 level PSO	6	2500

*All patients underwent bilateral conventional S2AI fixation supplemented with bilateral 8 mm × 80 mm UATS screws. PCO: Posterior column osteotomy, PSO: Pedicle subtraction osteotomy, EBL: Estimated blood loss.*

**Table 5:** Clinical results.

Patient No	Pre-Op VAS Lower Back	Last VAS Lower Back	Pre-Op VAS Sacro-Iliac	Last VAS Sacro-Iliac	Pre-Op ODI (%)	Last ODI (%)	Satisfaction
1	6	1	9	1	80	10	Extremely satisfied
2	8	1	9	2	90	20	Extremely satisfied
3	9	2	9	3	90	20	Extremely satisfied
4	4	1	8	1	60	5	Satisfied
5	5	1	9	2	90	10	Satisfied
6	8	1	8	2	70	20	Satisfied
7	8	2	9	2	70	10	Extremely satisfied
8	5	1	9	1	60	10	Extremely satisfied
9	8	1	2	2	80	20	Satisfied
10	6	2	8	2	60	20	Satisfied
11	8	3	8	2	90	20	Extremely satisfied
12	7	2	8	2	60	20	Extremely satisfied
13	9	2	9	3	80	10	Satisfied
14	8	1	8	2	80	20	Satisfied
15	7	1	8	2	60	10	Extremely satisfied
16	7	1	7	3	50	10	Satisfied
17	6	1	7	1	50	10	Satisfied
18	9	2	9	2	90	30	Extremely satisfied
19	9	3	9	4	90	30	Extremely satisfied
20	7	2	8	3	60	10	Satisfied
21	6	3	7	4	40	30	Satisfied
22	8	1	9	2	60	20	Extremely satisfied
23	7	3	7	2	40	10	Satisfied
24	8	3	8	4	40	10	Satisfied
25	6	3	6	4	40	10	Satisfied
26	8	3	9	4	90	40	Satisfied

*VAS: Visual analog scale, ODI: Oswestry Disability Index.*

**Table 6:** Spinopelvic alignment parameters.

Patient No	Pre-Op PI	Pre-Op LL	Pre-Op PI-LL	Post-Op LL	Post-Op PI-LL	Pre-Op SVA (cm)	Last SVA (cm)	Cobb Correction
1	55	37	18	48	7	4	4	None
2	60	55	5	63	−3	3	3	17° (70%)
3	62	44	18	53	9	6.5	4	14° (70%)
4	60	42	18	50	10	5	4.5	None
5	62	35	27	55	7	5	3	12° (75%)
6	54	41	13	54	0	4	4.5	None
7	60	40	20	50	10	7.5	4.5	8° (70%)
8	61	35	26	45	16	8.5	7.5	None
9	60	40	20	48	12	5.5	6.5	None
10	61	50	11	52	9	5	6	None
11	65	45	20	55	10	6	5	None
12	55	40	15	41	14	5.5	5	None
13	50	40	10	40	10	5	5	None
14	60	40	20	50	10	6.6	5	10° (95%)
15	62	45	17	56	6	5	4.5	None
16	50	30	20	39	11	6.5	5.5	None
17	52	38	14	63	−11	6	5	None
18	55	35	20	45	10	6.5	5	15° (90%)
19	58	45	13	47	11	7	6	10° (100%)
20	50	38	12	42	8	5	5	None
21	60	45	15	50	10	5.5	5	20° (100%)
22	61	42	19	50	11	7	5.5	None
23	70	48	22	58	12	6	5	None
24	50	38	12	42	8	5.5	5	None
25	48	35	13	38	10	5.5	5.5	None
26	75	35	40	40	35	8.23	8.08	None

*PI: Pelvic incidence, LL: Lumbar lordosis, SVA: Sagittal vertical axis. PI–LL: pelvic incidence-lumbar lordosis mismatch.*

**Table 7:** Cohort-level summary of clinical and radiographic outcomes.

Characteristic/Outcome	Cohort-Level Result
Patients	26
Age	69.8 ± 8.1 years
Sex	21 female (80.8%); 5 male (19.2%)
Surgical status	25 revision (96.2%); 1 index (3.8%)
Final follow-up	14.7 months; range 14–18 months
Lower-back VAS	7.2 ± 1.4 preoperatively → 1.8 ± 0.8 at final follow-up
SIJ-related VAS	8.0 ± 1.5 preoperatively → 2.4 ± 1.0 at final follow-up
ODI	68.1 ± 18.1% preoperatively → 16.7 ± 8.6% at final follow-up
Fusion assessment	CT in all 26 patients: early postoperative and 12 months; 23 met CT criteria for solid L5–S1 fusion, 3 indeterminate
Peri-screw radiolucency	Primary S2AI halo: 4/26 (15.4%); UATS halo: 1/26 (3.8%); descriptive only
Screw migration/rod fracture	0/0
Surgical-site infection	2/26 (7.7%); IV antibiotics; no debridement or implant removal
Reoperation for any cause	0/26
New neurological/vascular complication	0/0

## Data Availability

Additional data may be requested from the corresponding author upon reasonable request.

## Abbreviations

The following abbreviations are used in this manuscript:

S2AI	S2–alar–iliac
SIJ	Sacroiliac joint
UATS	uncoupled auxiliary trans-sacroiliac
FEA	Finite Element Analysis
RP	Reference Point
RBE2	Rigid Body Element 2
DOF	Degrees of Freedom
FABER	Flexion, Abduction and External Rotation
VAS	Visual Analogue Scale
ODI	Oswestry Disability Index
CT	Computed Tomography
BMD	Bone Mineral Density
PSO	Pedicle Subtraction Osteotomy
EBL	Estimated Blood Loss
PI-LL	Pelvic Incidence–Lumbar Lordosis
